# Modafinil and its metabolites enhance the anticonvulsant action of classical antiepileptic drugs in the mouse maximal electroshock-induced seizure model

**DOI:** 10.1007/s00213-015-3884-3

**Published:** 2015-02-21

**Authors:** Dorota Zolkowska, Marta Andres-Mach, Thomas E. Prisinzano, Michael H. Baumann, Jarogniew J. Luszczki

**Affiliations:** 1 Department of Neurology, School of Medicine, University of California, Davis, Sacramento, California USA; 2 Isobolographic Analysis Laboratory, Institute of Rural Health, Lublin, Poland; 3 Department of Medicinal Chemistry, The University of Kansas, Lawrence, Kansas USA; 4 Medicinal Chemistry Section, Intramural Research Program (IRP), NIDA, NIH, Baltimore, MD USA; 5 Department of Pathophysiology, Medical University of Lublin, Ceramiczna 1, 20-150 Lublin, Poland

**Keywords:** Antiepileptic drugs, Maximal electroshock-induced seizures, Pharmacokinetic/pharmacodynamic interaction, Modafinil, GBR 12909

## Abstract

**Rationale:**

Seizures occur when the excitability of brain circuits is not sufficiently restrained by inhibitory mechanisms. Although modafinil is reported to reduce GABA-activated currents and extracellular GABA levels in the brain, the drug exerts anticonvulsant effects in animal studies.

**Objectives:**

The aim of this study was to determine the effects of modafinil and its metabolites (sulfone and carboxylic acid) on the anticonvulsant action of four classical antiepileptic drugs (AEDs)—carbamazepine (CBZ), phenobarbital (PB), phenytoin (PHT), and valproate (VPA).

**Methods:**

Anticonvulsant activity was assessed with the maximal electroshock seizure threshold (MEST) test and MES test in mice. Brain concentrations of AEDs were measured to ascertain any pharmacokinetic contribution to the observed anticonvulsant effects.

**Results:**

Intraperitoneal injection of 75 mg kg^−1^ of modafinil or its metabolites significantly elevated the threshold for electroconvulsions in mice, whereas 50 mg kg^−1^ of each compound enhanced the anticonvulsant activity of CBZ, PHT, and VPA, but not that of PB. A 25-mg kg^−1^ dose of modafinil or its sulfone metabolite enhanced anticonvulsant activity of VPA. Modafinil and its metabolites (50 mg kg^−1^) did not alter total brain concentrations of PB and VPA but did elevate CBZ and PHT.

**Conclusions:**

Enhancement of anticonvulsant actions of VPA by modafinil in the mouse MES model is a pharmacodynamic effect. Collectively, our data suggest that modafinil may be a safe and beneficial adjunct to the therapeutic effects of AEDs in human patients.

## Introduction

Modafinil (diphenylmethylsulfinylacetamide) is wake-promoting medication approved for the treatment of narcolepsy, shift work sleep problems, and obstructive sleep apnea (Ballon and Feifel 2006; Minzenberg and Carter 2008). In the past decade, there has been a marked increase in off-label use of modafinil for a variety of indications (Peñaloza et al. 2013). As a nonamphetamine stimulant with low abuse liability, modafinil may be a safe alternative for treatment of fatigue syndrome and psychiatric disorders such as treatment-resistant depression and attention deficit/hyperactivity disorder (Minzenberg and Carter 2008; Swanson et al. 2006). Despite the widespread clinical use of modafinil, the precise mechanisms underlying its therapeutic efficacy are complex and not well understood (for review, see Ballon and Feifel 2006; Minzenberg and Carter 2008).

Modafinil is reported to affect many central neurotransmitter systems: dopamine, norepinephrine, 5-hydroxytryptamine, glutamate, GABA, histamine, and orexin. Moreover, it is suggested that excitatory effects of modafinil may be due to widespread disinhibition of excitatory networks caused by increased electrical coupling and decreased input resistance among electrically coupled neurons (Garcia-Rill et al. 2007). Initial preclinical studies indicated that stimulant effects of modafinil are distinct from those of amphetamine and may not involve dopamine systems (Duteil et al. 1990; Simon et al. 1995). However, more recent studies show that modafinil interacts with dopamine transporter (DAT) proteins to block dopamine uptake and elevate extracellular dopamine in the central nervous system of rats and in humans (Volkow et al. 2009; Zolkowska et al. 2009). Nevertheless, modafinil has a number of nondopaminergic effects which include activation of α1 adrenergic receptors (Duteil et al. 1990), enhancement of 5-hydroxytryptamine function (Ferraro et al. 2000), inhibition of GABA release (Ferraro et al. 1997, 1998), and stimulation of glutamate and histamine release (Ferraro et al. 1999; Ishizuka et al. 2003)

Even though modafinil is reported to reduce GABA-activated currents and extracellular GABA levels in different brain regions, the drug exerts antiepileptic effects (Chen et al. 2007). Specifically, modafinil is able to reduce seizure activity in the maximal electroshock (MES) model and in the pentylenetetrazole (PTZ)-kindling model. Surprisingly, the sulfone and acid metabolites of modafinil are reported to exert anticonvulsant activity in the MES model (Chatterjie et al. 2004).

It is generally accepted that seizures occur when the excitability of brain circuits is not sufficiently restrained by inhibitory mechanisms (Nadler 2012). The neurotransmitter changes associated with epileptic foci include decreased GABA activity mainly at GABA-A receptors and altered activity at calcium, sodium, chloride, and potassium channels. Increased intracellular and decreased extracellular calcium concentrations, as well as decreased GABA-ergic presynaptic inhibition, are important factors in the hyperexcitability that contributes in epileptogenesis.

The aim of our study was to further explore the effects of modafinil and its two metabolites, diphenylmethylsufonylacetamide (i.e., sulfone metabolite) and diphenylmethylthioacetic acid (i.e., acid metabolite), on the threshold for electroconvulsions and on the protective activity of four classical antiepileptic drugs (AEDs): carbamazepine (CBZ), phenobarbital (PB), phenytoin (PHT), and valproate (VPA) in the mouse MES-induced seizure model. Additionally, because modafinil is a DAT blocker, we wished to compare the anticonvulsant effects of modafinil to the prototypical DAT blocker 1-[2-[bis(4-fluorophenyl)methoxy]ethyl]-4-(3-phenylpropyl)piperazine (GBR 12909). The threshold for electroconvulsions and the MES test are established experimental models of tonic-clonic seizures and, to a certain extent, of partial convulsions with or without secondary generalization in humans (Löscher et al. 1991). Both tests are used as standard screening procedures for identifying new anticonvulsant agents or treatments which can be combined with classical AEDs (Löscher et al. 1991). Therefore, these in vivo seizure models were selected for evaluation of the effects of modafinil and other test drugs alone as well as in combination with classical AEDs. Finally, total brain AED concentrations were measured with a fluorescence polarization immunoassay to ascertain whether effects of modafinil or its metabolites were related to altered pharmacokinetics of AEDs.

## Materials and methods

### Animals and experimental conditions

Adult male Swiss mice (weighing 22–26 g) were kept in colony cages with free access to food and tap water, housed under standardized housing conditions (natural light-dark cycle, temperature of 23 ± 1 °C, relative humidity of 55 ± 5 %). After 7 days of adaptation to laboratory conditions, animals were randomly assigned to experimental groups each composed of eight mice. Each mouse was used only once, and all tests were performed between 08:00 a.m. and 03:00 p.m. Experiments were performed after a minimum 30-min period of acclimation to the experimental room. Procedures involving animals and their care were conducted in accordance with current European Community and Polish legislation on animal experimentation. Additionally, all efforts were made to minimize animal suffering and to use only the number of animals necessary to produce reliable scientific data. The experimental protocols and procedures described in this manuscript were approved by the Second Local Ethics Committee at the University of Life Sciences in Lublin (license nos. 3/2011, 5/2012, 16/2012) and complied with the European Communities Council Directive of 24 November 1986 (86/609/EEC).

### Drugs

The following drugs were used: modafinil, diphenylmethylsufonylacetamide (sulfone metabolite), diphenylmethylthioacetic acid (acid metabolite), and 1-[2-[bis(4-fluorophenyl)methoxy]ethyl]-4-(3-phenylpropyl)piperazine HCl (GBR 12909) were synthesized by Dr. T. E. Prisinzano (Department of Medicinal Chemistry, The University of Kansas, Lawrence KS, USA), carbamazepine (CBZ—a gift from Polpharma, Starogard Gdański, Poland), phenobarbital (PB—Polfa, Kraków, Poland), phenytoin (PHT—Polfa, Warszawa, Poland), and valproate (VPA—magnesium salt—kindly donated by ICN-Polfa S.A., Rzeszów, Poland). All drugs, except for VPA and GBR 12909, were suspended in an aqueous 1 % solution of Tween 80 (Sigma, St. Louis, MO, USA); VPA and GBR 12909 were directly dissolved in distilled water. All drugs were administered intraperitoneally (i.p.), in a volume of 10-ml kg^−1^ body weight, at the following pretreatment times: PHT 120 min, PB 60 min, modafinil, its metabolites, GBR 12909, CBZ and VPA 30 min before electroconvulsions, and brain sampling for the measurement of AED concentrations. The pretreatment times before testing of the AEDs were based upon information about their biological activity from the literature (Löscher et al. 1991) and our previous experiments (Luszczki et al. 2009a, b, 2010a, b). The pretreatment time (30 min) before testing modafinil, sulfone, and acid metabolites, or GBR 12909 were based on our previous experiments and literature data (Chatterjie et al. 2004; Zolkowska et al. 2009).

### Electroconvulsions

Electroconvulsions were induced by applying an alternating current (50 Hz; 500 V) via ear clip electrodes from a rodent shocker generator (type 221; Hugo Sachs Elektronik, Freiburg, Germany). The stimulus duration was 0.2 s. Tonic hind limb extension was used as the endpoint. This apparatus was used to induce seizures in two methodologically different experimental approaches: maximal electroshock seizure threshold (MEST) test and MES test (Löscher et al. 1991).

### Maximal electroshock seizure threshold (MEST) test

The MEST test was first used to assess the anticonvulsant effects of modafinil, sulfone, and acid metabolites or GBR 12909 administered alone. In this test, at least four groups of control mice, each consisting of eight animals, were challenged with currents of varying intensities ranging between 5 and 8 mA so that 10–30, 30–50, 50–70, and 70–90 % of animals exhibited the endpoint. After establishing the current intensity-effect curve (i.e., current intensity in mA vs percentage of mice convulsing) for each dose of modafinil, its metabolites, or GBR 12909, the electroconvulsive threshold was calculated according to the log-probit method of Litchfield and Wilcoxon (1949). The electroconvulsive threshold was expressed as the median current strength value (CS_50_ in mA) predicted to produce tonic hind limb extension in 50 % of the animals tested. This experimental procedure was performed for various increasing doses of modafinil and sulfone and acid metabolites (12.5–75 mg kg^−1^) or GBR 12909 (6.25–50 mg kg^−1^), until the thresholds for electroconvulsions of tested compounds were statistically different from that of the control animals. Only doses of modafinil, sulfone, and acid metabolite or GBR 12909 that did not significantly affect the seizure threshold in the MEST test were selected for testing in combination with four classical AEDs in the MES test (see below). This approach allowed us to rule out any contribution of the intrinsic anticonvulsant efficacy of tested substances in the effects observed in combination with the AEDs in the MES test. Subsequently, the percentage increase in CS_50_ values for animals injected with increasing doses of modafinil, sulfone, and acid metabolites and GBR 12909 over the control (vehicle-treated animals) was calculated. The doses of modafinil, its metabolites and GBR 12909, and their resultant percentage of threshold increase over the control (vehicle-treated animals) were graphically plotted in rectangular coordinates of the Cartesian plot system and examined with least-squares linear regression analysis. From the linear regression equation, the TID_20_ values were determined, as recommended by Löscher et al. (1991) and Swinyard et al. (1952). This experimental procedure has been described in more detail in our earlier studies (Luszczki and Czuczwar 2005, 2007; Luszczki et al. 2013).

### Maximal electroshock seizure (MES) test

In the MES test, mice were challenged with a current of the fixed intensity (25 mA) that was 4–5-fold higher than the CS_50_ value in vehicle-treated control mice (Löscher et al. 1991). These parameters of stimulation (maximal electroshock) typically result in all mice responding with tonic hind limb extension immediately after stimulation. The AEDs administered alone and their combination with modafinil, its metabolites, or GBR 12909 were tested for their ability to increase the number of animals not responding with tonus (i.e., protected from tonic hind limb extension) after stimulation. Again, at least four groups of mice, each consisting of eight animals and treated with a different dose of the AEDs alone or in combination with modafinil, sulfone and acid metabolites or GBR 12909, were challenged with a current of 25 mA to yield 10–30, 30–50, 50–70, and 70–90 % of animals protected from tonic seizures. After constructing a dose-effect curve (i.e., dose in mg kg^−1^ vs percentage of mice protected), the protective median effective dose (ED_50_) value of the AED tested was calculated according to a log-probit method by Litchfield and Wilcoxon (1949). Each ED_50_ value represented a dose of the AED (in mg kg^−1^) predicted to protect 50 % of mice tested against MES-induced extension of the hind limbs. Modafinil, its two metabolites, and GBR 12909 were tested for their ability to affect the anticonvulsive potency of AEDs. As mentioned earlier, modafinil, sulfone, and acid metabolites or GBR 12909 were administered in doses that per se had no effect on seizure threshold in the MEST test. In this experimental protocol, an increase in the anticonvulsant potency of the AED tested in combination with modafinil, sulfone, and acid metabolites or GBR 12909 would be reflected by a lower ED_50_ value of the test AED (i.e., lower dose of test drug was necessary to protect 50 % of mice challenged). In the present study, CBZ was administered at doses ranging between 2 and 18 mg kg^−1^, PB at doses ranging between 10 and 35 mg kg^−1^, PHT at doses ranging between 4 and 14 mg kg^−1^, and VPA at doses ranging between 175 and 400 mg kg^−1^. These AED doses suppressed tonic seizures in 10–90 % of mice subjected to the MES test.

### Measurement of total brain antiepileptic drug concentrations

Pharmacokinetic evaluation of total brain AED concentrations was performed for the combinations of modafinil, sulfone, and acid metabolites or GBR 12909 with CBZ, PB, PHT, and VPA (at the doses that corresponded to their ED_50_ values from the MES test). Specifically, mice pretreated with a given AED alone or in combination with modafinil, sulfone, and acid metabolites or GBR 12909 were decapitated at times reflecting the peak of maximum anticonvulsant effects for the drugs in the MES test. The whole brains of mice were removed from the skulls, weighed, harvested, and homogenized using Abbott buffer (1:2 weight/volume; Abbott Laboratories, North Chicago, IL, USA) in an Ultra-Turrax T8 homogenizer. The homogenates were then centrifuged at 10,000 *g* for 10 min, and the supernatant samples of 100 μl were collected and then analyzed for AED content. Total brain concentrations of CBZ, PB, PHT, and VPA were measured by a fluorescence polarization immunoassay using an analyzer (Abbott TDx) and manufacturer-supplied reagent kits (Abbott Laboratories, North Chicago, IL, USA). Total brain AED concentrations are expressed in μg g^−1^ of wet brain tissue as means ± standard error of the mean (S.E.M.) of at least eight separate brain preparations.

### Step-through passive avoidance task

The effects of combinations of modafinil (50 mg kg^−1^), its metabolites (50 mg kg^−1^), or GBR 12909 (25 mg kg^−1^) with different classical AEDs were quantified by the step-through passive avoidance task of Venault et al. (1986). AEDs were administered at doses corresponding to their ED_50_ values from the MES test. Each animal received an AED either alone or in combination either with modafinil, sulfone, and acid metabolites or with GBR 12909 on the first day before training. The time before commencement of the training session (after drug administration) in the step-through passive avoidance task was identical to that for the MES test. Subsequently, the animals were placed in an illuminated box (10 × 13 × 15 cm) connected to a larger dark box (25 × 20 × 15 cm) equipped with an electric grid floor. Entrance of the animals to the dark box was punished by an adequate electric footshock (0.6 mA for 2 s). The animals that did not enter the dark compartment were excluded from subsequent experimentation. On the following day (24 h later), the pretrained animals were placed again into the illuminated box and observed for up to 180 s. Mice that avoided the dark compartment for 180 s were considered as having remembered the task. The time the mice took to enter the dark box was noted, and the median latencies (retention times) with 25th and 75th percentiles were calculated.

### Grip strength test

The effects of combinations of modafinil (50 mg kg^−1^), sulfone and acid metabolites (50 mg kg^−1^), or GBR 12909 (25 mg kg^−1^) with different classical AEDs at doses corresponding to their ED_50_ values from the MES test on skeletal muscular strength in mice were quantified by the grip strength test of Meyer et al. (1979). The grip strength apparatus (BioSeb, Chaville, France) comprised a wire grid (8 × 8 cm) connected to an isometric force transducer (dynamometer). The mice were lifted by the tails so that their forepaws could grasp the grid. The mice were then gently pulled backward by the tail until the grid was released. The maximal force exerted by the mouse before losing grip was recorded. The mean of three measurements for each animal was calculated, and subsequently, the mean maximal force of eight animals per group was determined. The muscular strength in mice is expressed in N (newtons) as the means ± S.E.M. of at least eight determinations.

### Chimney test

The chimney test of Boissier et al. (1960) was used to quantify the adverse effect potential of classical AEDs administered in combination with modafinil, its metabolites, or GBR 12909. In this test, the animals had to climb backwards up a plastic tube (3-cm inner diameter, 30 cm long), and impairment of motor performance was indicated by the inability of the mice to climb backward up the transparent tube within 60 s. The acute adverse effect potentials for the combinations of classical AEDs with either modafinil, sulfone, and acid metabolites or GBR 12909 were determined for the AEDs administered at doses corresponding to their ED_50_ values from the MES test when combined with modafinil (50 mg kg^−1^), its metabolites (50 mg kg^−1^), or GBR 12909 (25 mg kg^−1^).

### Statistics

Both CS_50_ and ED_50_ values with their 95 % confidence limits were calculated by computer log-probit analysis according to Litchfield and Wilcoxon (1949). Subsequently, the respective 95 % confidence limits were transformed to S.E.M. as described previously (Luszczki et al. 2009a). Statistical analysis of data from the MEST test was performed with one-way analysis of variance (ANOVA) followed by the post hoc Tukey-Kramer test for multiple comparisons among four CS_50_ values. Statistical analysis of data from the MES test was performed with one-way ANOVA followed by the post hoc Tukey-Kramer test for multiple comparisons among three ED_50_ values. Total brain AED concentrations were statistically compared using the unpaired Student’s *t* test. The results obtained in the step-through passive avoidance task were statistically evaluated using Kruskal-Wallis nonparametric ANOVA. The results from the grip strength test were verified with one-way ANOVA. The data from the chimney test were statistically analyzed with the Fisher’s exact probability test. Differences among values were considered statistically significant if *P* < 0.05. All statistical tests were performed using GraphPad Prism version 5.0 for Windows (GraphPad Software, San Diego, CA, USA).

## Results

### Influence of modafinil, sulfone, and acid metabolites or GBR 12909 on the threshold for electroconvulsions

**Fig. 1:**
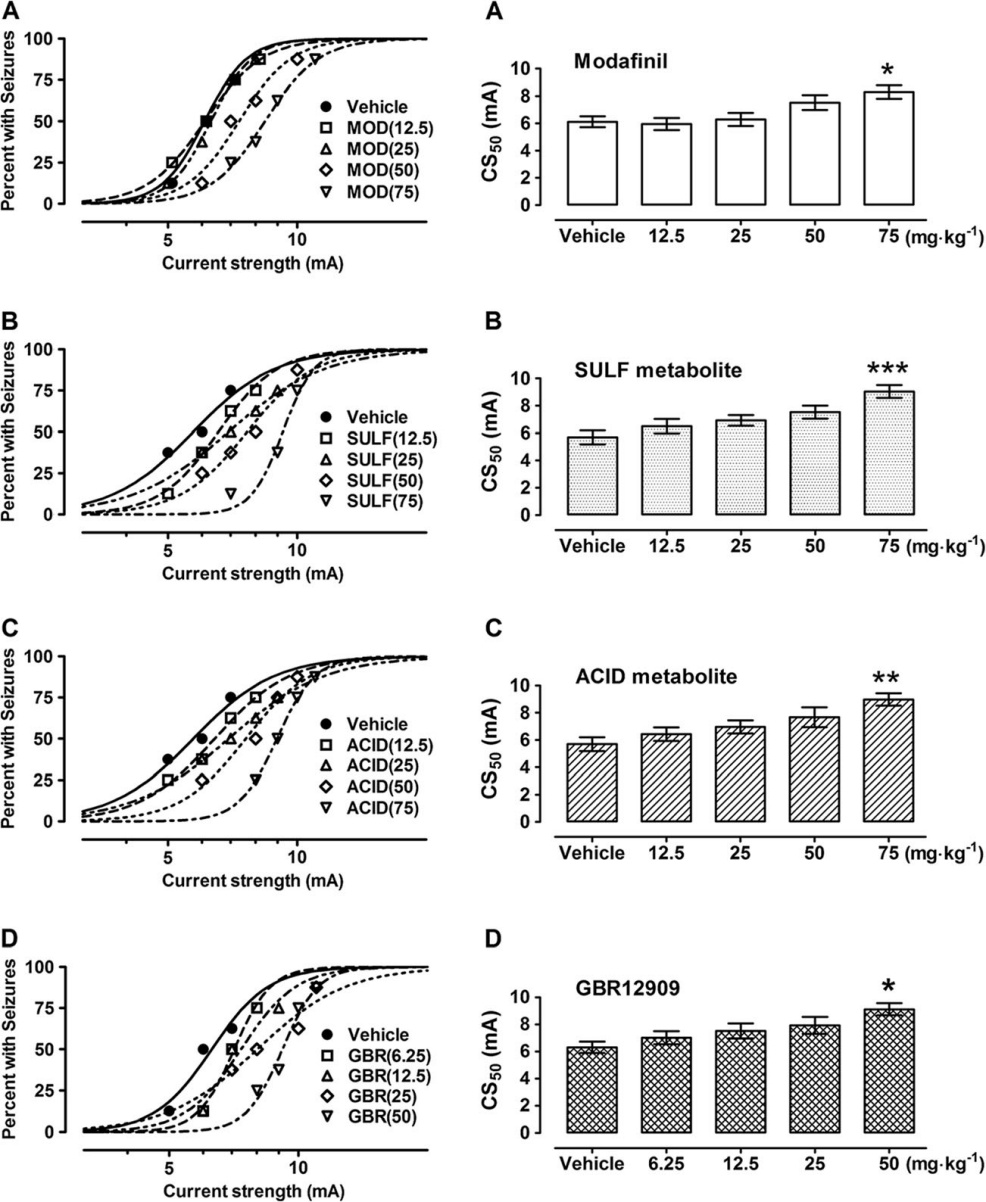
**a–d** Effect of modafinil (MOD) (**a**), its sulfone (SULF) (**b**) and acid (ACID) (**c**) metabolites, and GBR12909 (GBR) (**d**) on the threshold test for electroconvulsions (MEST) in mice. *Left panel* graphs illustrate current intensity–response relationships for tonic hind limb extension in the threshold test for electroconvulsions in mice for MOD, SULF, ACID, and GBR. All tested drugs were administered i.p. at 30 min before electroconvulsions. Data points indicate percentage of animals protected. Each point represents eight mice. *Right panel* columns represent median current strengths (CS_50_ in mA ± S.E.M.) for modafinil (**a**), SULF metabolite (**b**), ACID metabolite (**c**), and GBR12909 (**d**), required to produce tonic hind limb extension in 50 % of animals tested. Statistical analysis of data was performed with one-way ANOVA followed by the post hoc Tukey-Kramer test for multiple comparisons. *P < 0.05, **P < 0.01, and ***P < 0.001 vs the respective control group (vehicle-treated animals)

Modafinil and its two metabolites administered at a dose of 75 mg kg^−1^ i.p. significantly increased the threshold for electroconvulsions in mice (Table 1). The thresholds were elevated from 6.12 ± 0.40 to 8.29 ± 0.48 mA (*F*[4,99] = 4.70; *P* = 0.002), from 5.69 ± 0.51 to 9.04 ± 0.47 mA (*F*[4,115] = 5.49; *P* = 0.0004), and from 5.69 ± 0.51 to 8.98 ± 0.45 mA (*F*[4,189] = 4.414; *P* = 0.0022) by modafinil and its sulfone and acid metabolites, respectively (Table 1, Fig. 1a–d). The experimentally derived CS_50_ values for animals receiving test compounds at doses 12.5–50 mg kg^−1^ did not significantly differ from that for control animals subjected to the MEST test (Table 1, Fig. 1a–d). Additionally, because modafinil is a DAT blocker, we compared the anticonvulsant effects of modafinil and its two metabolites to the prototypical DAT blocker—GBR 12909. GBR 12909 administered at 50 mg kg^−1^ i.p. significantly elevated the threshold for electroconvulsions in mice from 6.31 ± 0.43 to 9.12 ± 0.45 mA (*F*[4,107] = 3.14; *P* = 0.02) while the doses of 6.25–25 mg kg^−1^ were ineffective (Table 1, Fig. 1a–d).

**Table 1:** Influence of modafinil (MOD), sulfone (SULF), and acid (ACID) derivatives of MOD and GBR12909 on the threshold for electroconvulsions in mice

Treatment (mg kg^−1^)	CS_50_ (mA)	*n*	TI (%)
Vehicle	6.12 ± 0.40	16	–
MOD (12.5)	5.95 ± 0.44	24	−2.78
MOD (25)	6.29 ± 0.48	16	2.78
MOD (50)	7.52 ± 0.54	24	22.88
MOD (75)	8.29 ± 0.48*	24	35.46
*F* (4; 99) = 4.702; *P* = 0.0016			
Vehicle	5.69 ± 0.51	24	–
SULF (12.5)	6.50 ± 0.52	24	14.24
SULF (25)	6.92 ± 0.40	32	21.62
SULF (50)	7.53 ± 0.48	24	32.34
SULF (75)	9.04 ± 0.47***	16	58.88
*F* (4; 115) = 5.494; *P* = 0.0004			
Vehicle	5.69 ± 0.51	24	–
ACID (12.5)	6.42 ± 0.50	32	12.83
ACID (25)	6.96 ± 0.48	32	22.32
ACID (50)	7.67 ± 0.72	32	34.80
ACID (75)	8.98 ± 0.45**	24	57.82
*F* (4; 189) = 4.414; *P* = 0.0022			
Vehicle	6.31 ± 0.43	16	–
GBR (6.25)	7.02 ± 0.48	16	11.25
GBR (12.5)	7.52 ± 0.55	24	19.18
GBR (25)	7.93 ± 0.62	32	25.67
GBR (50)	9.12 ± 0.45*	24	44.53
*F* (4; 107) = 3.135; *P* = 0.0176			

Results are presented as median current strengths (CS_50_ in mA ± S.E.M.) required to produce tonic hind limb extension in 50 % of animals tested. MOD, SULF, ACID, and GBR were administered i.p. at 30 min before electroconvulsions. Statistical analysis of data was performed with one-way ANOVA followed by the post hoc Tukey-Kramer test for multiple comparisons. Additionally, the threshold for control animals was considered as a baseline (reference) value, allowing for the subsequent calculation of a percentage of threshold increase (TI) in animals after MOD, SULF, ACID, and GBR administration. *n* number of animals tested at those current strength intensities, whose seizure effects ranged between 16 and 84 % according to Litchfield and Wilcoxon (1949)

*F* F-statistics from one-way ANOVA, *P* probability from one-way ANOVA

*P < 0.05, **P < 0.01, and ***P < 0.001 vs the respective control group (vehicle-treated animals)

**Fig. 2:**
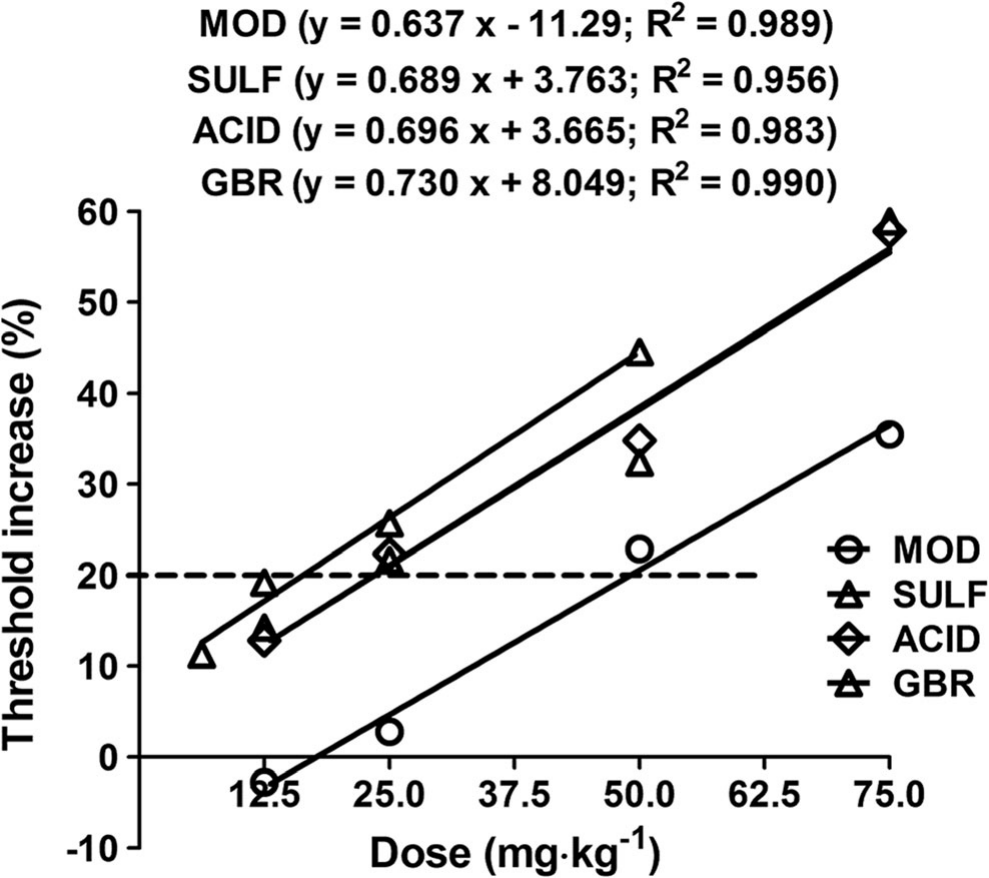
Dose-threshold increase relationship for modafinil (MOD), its sulfone (SULF) and acid (ACID) metabolites, and GBR12909 (GBR) in maximal electroshock seizure threshold (MEST) test in mice. Points placed on the graph represent threshold increasing doses of MOD, SULF, ACID, and GBR, experimentally denoted in the MEST test in mice. Linear regression analysis allowed determination of the equation for dose-threshold increase relationship for MOD, SULF, ACID, and GBR, as follows: y = 0.637 x − 11.29 (*R* ^2^ = 0.989) for MOD, y = 0.689 x + 3.763 (*R* ^2^ = 0.956) for SULF, y = 0.696 x + 3.665 (*R* ^2^ = 0.983) for ACID, and y = 0.730 x + 8.049 (*R* ^2^ = 0.990) for GBR, where y is the threshold increase in %, x is the drug dose, and *R* ^2^ is the coefficient of determination. From these equations, the TID_20_ (threshold increasing doses by 20 %) for the MEST test were calculated. In this study, these values were 49.12 mg kg^−1^ for MOD, 23.57 mg kg^−1^ for SULF, 23.47 mg kg^−1^ for ACID, and 16.37 mg kg^−1^ for GBR, respectively. The *dashed line* indicates threshold increase by 20 % and the respective doses of the tested compounds in the MEST test, which exerted this (20 %) effect. MOD, SULF, ACID, and GBR were administered systemically (i.p.), 30 min before the threshold evaluation

The equation for dose-threshold increase relationship for modafinil, its sulfone, and acid metabolites and GBR were as follows: y = 0.637 x − 11.29 (*R* ^2^ = 0.989) for modafinil, y = 0.689 x + 3.763 (*R* ^2^ = 0.956) for sulfone metabolite, y = 0.696 x + 3.665 (*R* ^2^ = 0.983) for acid metabolite, and y = 0.730 x + 8.049 (*R* ^2^ = 0.990) for GBR12909, where y is the threshold increase in %, x is the drug dose, and *R* ^2^ is the coefficient of determination. The experimentally derived TID_20_ (dose that increases threshold by 20 %) values were 49.12 mg kg^−1^ for modafinil, 23.57 mg kg^−1^ for sulfone metabolite, 23.47 mg kg^−1^ for acid metabolite, and 16.37 mg kg^−1^ for GBR12909, respectively, in the MEST test in mice (Fig. 2).

### Effects of modafinil, its metabolites, or GBR 12909 on the protective action of carbamazepine, phenobarbital, phenytoin, and valproate in the mouse maximal electroshock seizure model

**Fig. 3:**
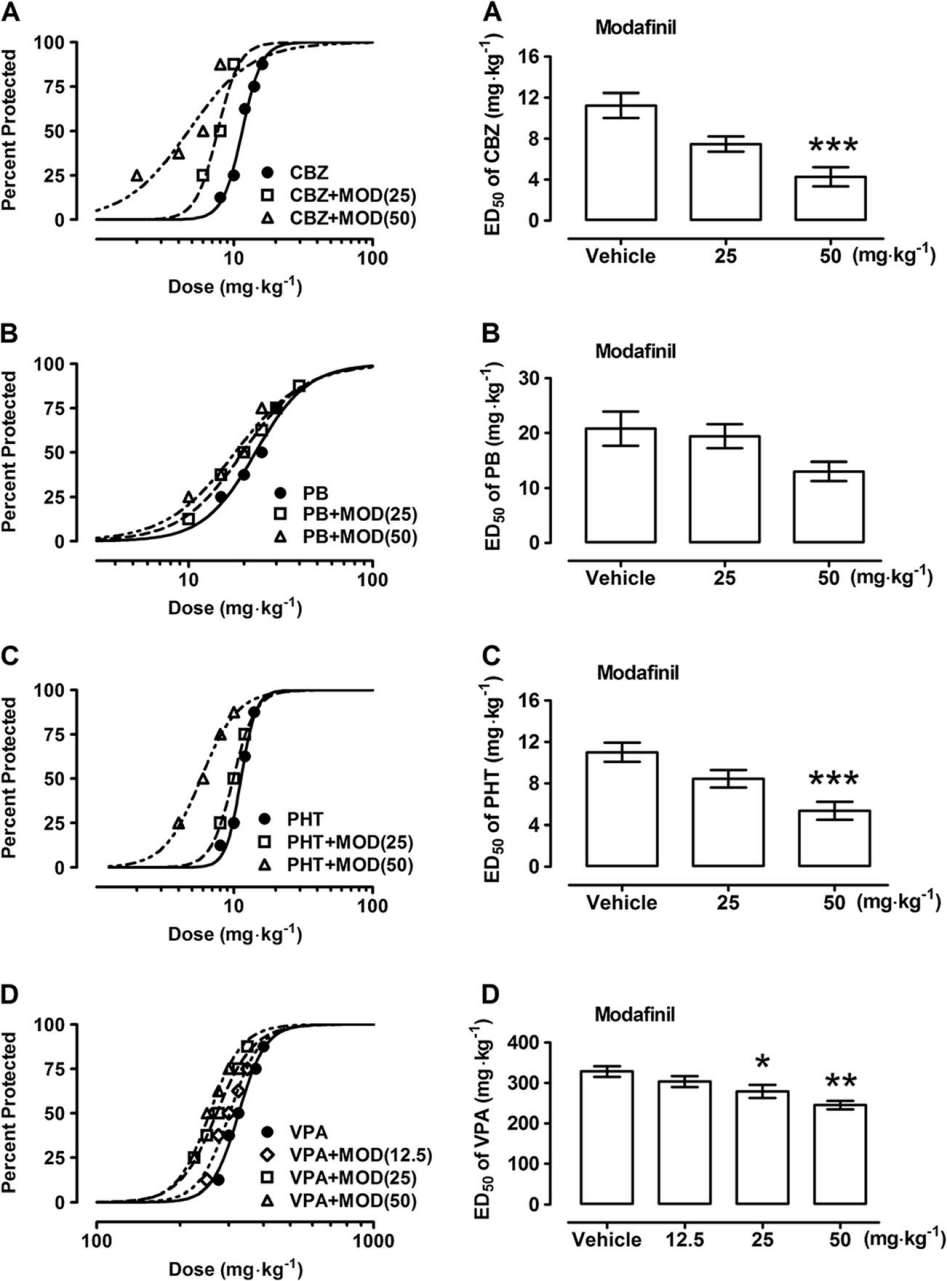
**a–d** Effects of modafinil (MOD) on the anticonvulsant action of carbamazepine (CBZ), phenobarbital (PB), phenytoin (PHT), and valproate (VPA) in the mouse maximal electroshock (MES)-induced seizure model. *Left panel* dose–response relationships for protective activity of classical antiepileptic drugs (AEDs) [CBZ (**a**), PB (**b**), PHT (**c**), and VPA (**d**)] alone and in combination with modafinil (MOD) in the mouse maximal electroshock (MES)-induced seizure model. The AEDs were administered i.p. CBZ 30 min, PB 60 min, PHT 120 min, and VPA 30 min prior to the MES test. MOD was administered i.p. at 30 min before the MES test. Data points indicate percentage of animals protected. Each point represents eight mice. *Right panel* columns represent median effective doses (ED_50_ in mg kg^−1^ ± S.E.M.) of AEDs [CBZ (**a**), PB (**b**), PHT (**c**), and VPA (**d**)], protecting 50 % of animals tested against MES-induced hind limb extension. Statistical analysis of data was performed with one-way ANOVA followed by the post hoc Tukey-Kramer test for multiple comparisons. *P < 0.05, **P < 0.01, and ***P < 0.001 vs the respective control group (an AED + vehicle-treated animals)

**Fig. 4:**
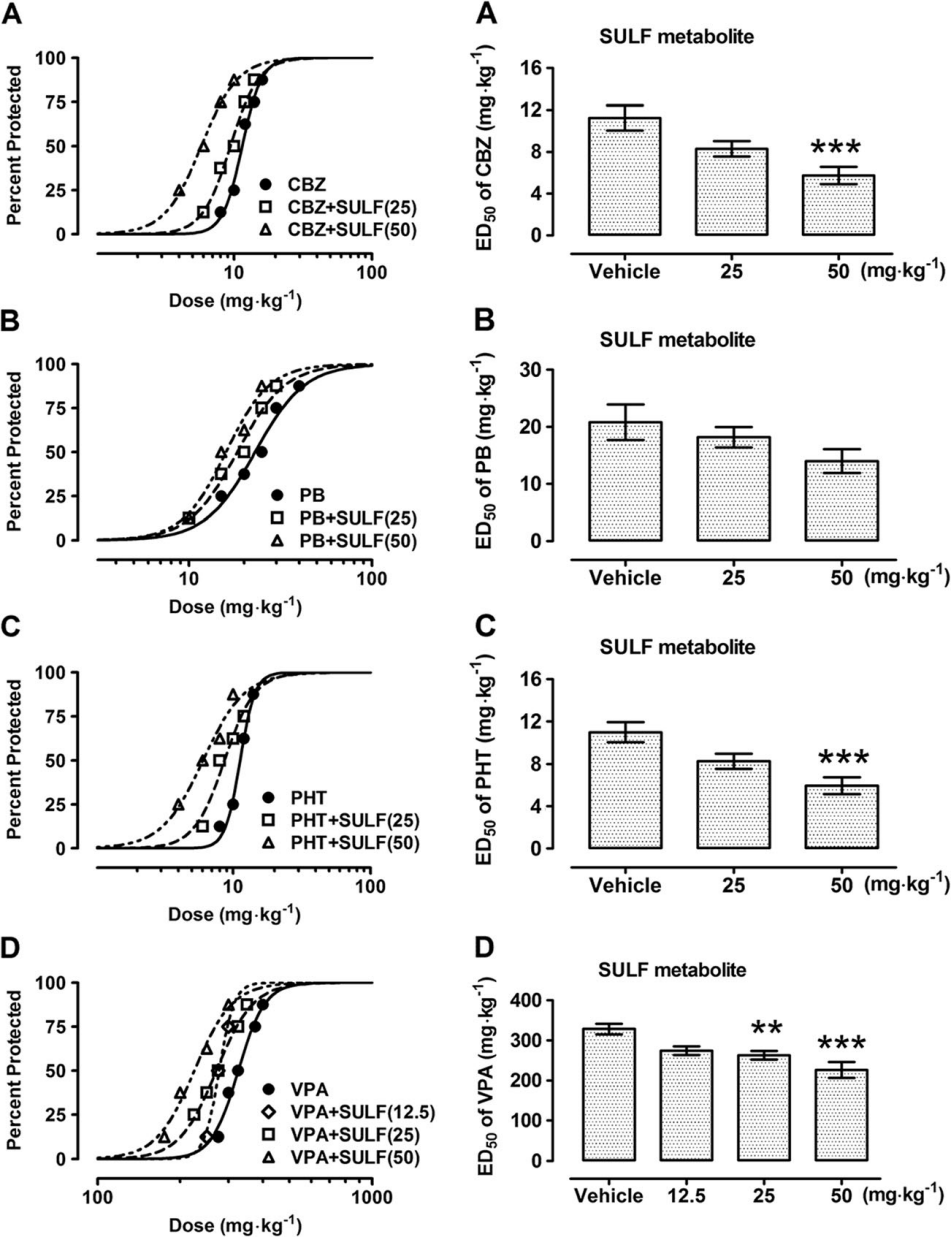
**a–d** Effects of sulfone (SULF) derivative of modafinil on the anticonvulsant action of carbamazepine (CBZ), phenytoin (PHT), phenobarbital (PB), and valproate (VPA) in the mouse maximal electroshock (MES)-induced seizure model. *Left panel* dose–response relationships for protective activity of classical antiepileptic drugs (AEDs) [CBZ (**a**), PB (**b**), PHT (**c**), and VPA (**d**)] alone and in combination with SULF derivative of modafinil in the mouse maximal electroshock (MES)-induced seizure model. The AEDs were administered i.p. CBZ 30 min, PB 60 min, PHT 120 min, and VPA 30 min prior to the MES test. SULF derivative of modafinil was administered i.p. at 30 min before the MES test. Data points indicate percentage of animals protected. Each point represents eight mice. *Right panel* columns represent median effective doses (ED_50_ in mg kg^−1^ ± S.E.M.) of AEDs [CBZ (**a**), PB (**b**), PHT (**c**), and VPA (**d**)], protecting 50 % of animals tested against MES-induced hind limb extension. The AEDs were administered i.p. CBZ 30 min, PB 60 min, PHT 120 min, and VPA 30 min prior to the MES test. SULF derivative of modafinil was administered i.p. at 30 min before the MES test. Statistical analysis of data was performed with one-way ANOVA followed by the post hoc Tukey-Kramer test for multiple comparisons. **P < 0.01 and ***P < 0.001 vs the respective control group (an AED + vehicle-treated animals)

**Fig. 5:**
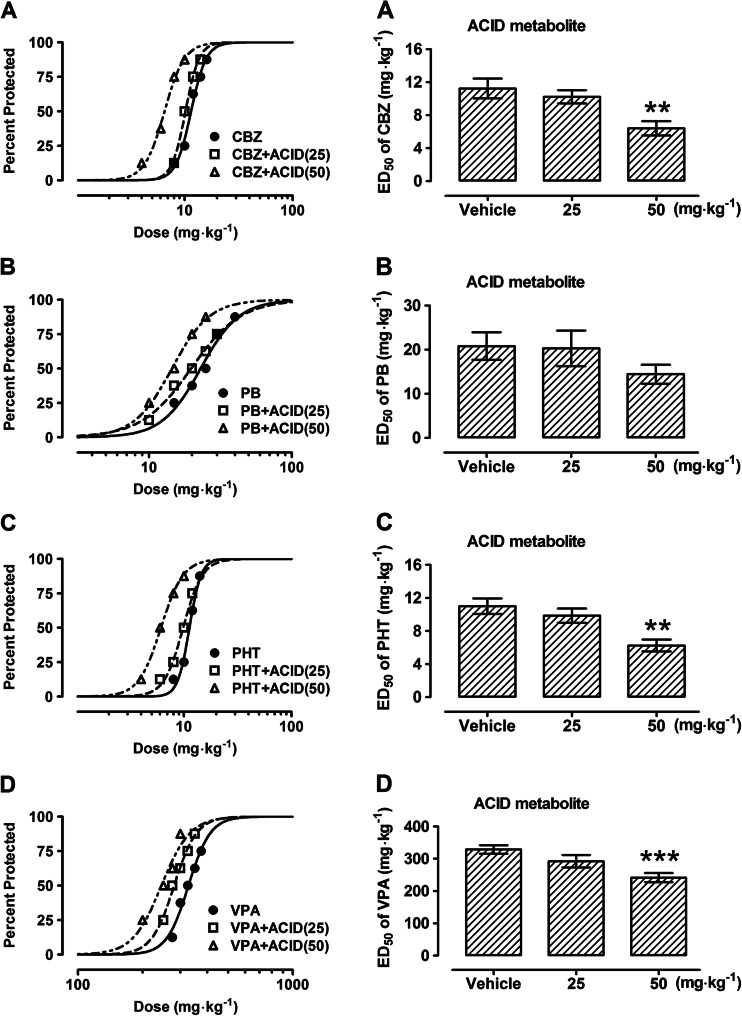
**a–d** Effects of acid (ACID) derivative of modafinil on the anticonvulsant action of carbamazepine (CBZ), phenytoin (PHT), phenobarbital (PB), and valproate (VPA) in the mouse maximal electroshock (MES)-induced seizure model. *Left panel* dose–response relationships for protective activity of classical antiepileptic drugs (AEDs) [CBZ (**a**), PB (**b**), PHT (**c**), and VPA (**d**)] alone and in combination with ACID derivative of modafinil in the mouse maximal electroshock (MES)-induced seizure model. The AEDs were administered i.p. CBZ 30 min, PB 60 min, PHT 120 min, and VPA 30 min prior to the MES test. ACID derivative of modafinil was administered i.p. at 30 min before the MES test. Data points indicate percentage of animals protected. Each point represents eight mice. *Right panel* columns represent median effective doses (ED_50_ in mg kg^−1^ ± S.E.M.) of AEDs, protecting 50 % of animals tested against MES-induced hind limb extension. Statistical analysis of data was performed with one-way ANOVA followed by the post hoc Tukey-Kramer test for multiple comparisons. **P < 0.01 and ***P < 0.001 vs the respective control group (an AED + vehicle-treated animals)

**Fig. 6:**
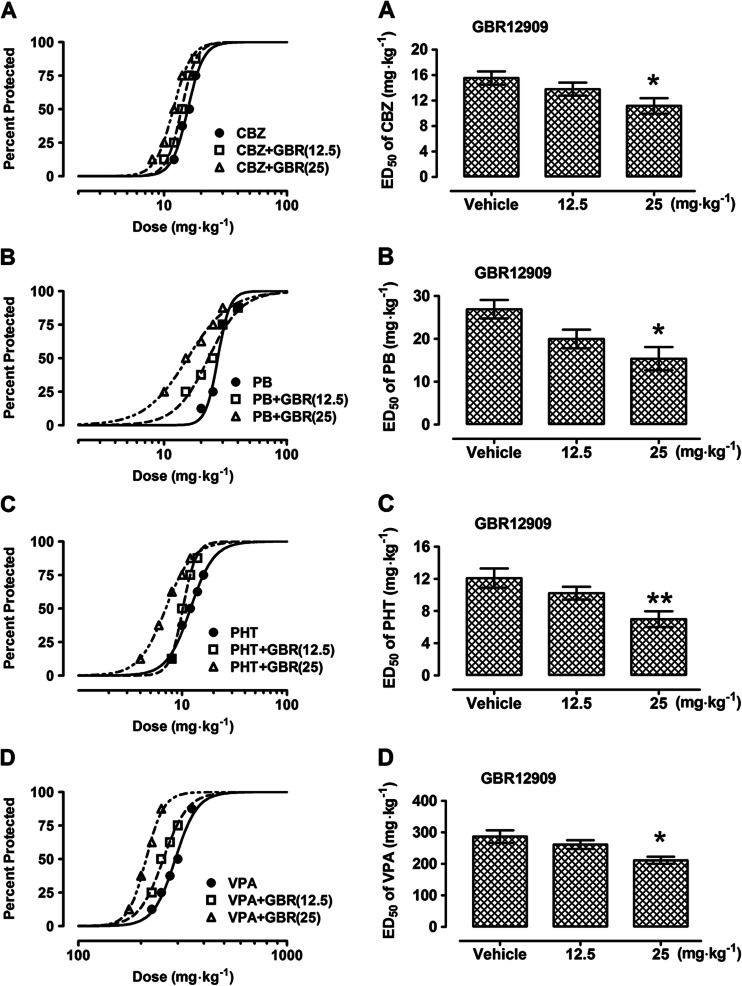
**a–d** Effects of GBR12909 (GBR) on the anticonvulsant action of carbamazepine (CBZ), phenobarbital (PB), phenytoin (PHT), and valproate (VPA) in the mouse maximal electroshock (MES)-induced seizure model. *Left panel* dose–response relationships for protective activity of classical antiepileptic drugs (AEDs) [CBZ (**a**), PB (**b**), PHT (**c**), and VPA (**d**)] alone and in combination with GBR12909 (GBR) in the mouse maximal electroshock (MES)-induced seizure model. The AEDs were administered i.p. CBZ 30 min, PB 60 min, PHT 120 min, and VPA 30 min prior to the MES test. GBR was administered i.p. at 30 min before the MES test. Data points indicate percentage of animals protected. Each point represents eight mice. *Right panel* columns represent median effective doses (ED_50_ in mg kg^−1^ ± S.E.M.) of AEDs [CBZ (**a**), PB (**b**), PHT (**c**), and VPA (**d**)], protecting 50 % of animals tested against MES-induced hind limb extension. Statistical analysis of data was performed with one-way ANOVA followed by the post hoc Tukey-Kramer test for multiple comparisons. *P < 0.05 and **P < 0.01 vs the respective control group (an AED + vehicle-treated animals)

All investigated classical AEDs (CBZ, PB, PHT, and VPA) administered alone exhibited a clear-cut anticonvulsant activity in the MES test in mice (Table 2, Fig. 3a–d). When modafinil (50 mg kg^−1^) was coadministered with CBZ, PHT, and VPA, it significantly enhanced the anticonvulsant action of AEDs in the MES test by reducing the ED_50_ value of CBZ from 11.23 ± 1.20 to 4.28 ± 0.93 mg kg^−1^ (*F*[2,61] = 11.95; *P* < 0.0001), PHT from 11.00 ± 0.93 to 5.35 ± 0.86 mg kg^−1^ (*F*[2,61] = 9.83; *P* = 0.0002), and VPA from 328.6 ± 13.17 to 245.3 ± 10.45 mg kg^−1^ (*F*[3,140] = 5.432; *P* = 0.002) (Table 2, Fig. 3a–d). Similarly, the sulfone and acid metabolites at a dose of 50 mg kg^−1^ significantly enhanced the anticonvulsant action of CBZ and VPA (Tables 3 and 4, Figs. 4a–d and 5a–d). The sulfone reduced the ED_50_ value of CBZ from 11.23 ± 1.20 to 5.74 ± 0.82 mg kg^−1^ (*F*[2,53] = 9.09; *P* = 0.0004) and PHT from 11.00 ± 0.93 to 5.95 ± 0.80 mg kg^−1^ (*F*[2,61] = 8.76; *P* = 0.0005), respectively (Table 3, Fig. 4a–d). The acid metabolite reduced the ED_50_ values of CBZ from 11.23 ± 1.20 to 6.40 ± 0.87 mg kg^−1^ (*F*[2,45] = 6.86; *P* = 0.003) and PHT from 11.00 ± 0.93 to 6.25 ± 0.71 mg kg^−1^ (*F*[2,53] = 7.24; *P* = 0.002), respectively (Table 4, Fig. 5a–d). Neither modafinil nor its metabolites at the dose of 25 mg kg^−1^ had an impact on the anticonvulsant action of CBZ or PHT against MES-induced seizures in mice (Tables 2, 3 and 4, Figs. 3a–d, 4a–d, and 5a–d). However, modafinil and its sulfone metabolite at doses of 25 and 50 mg kg^−1^, but not at the dose of 12.5 mg kg^−1^, significantly enhanced the anticonvulsant action of VPA (Tables 2 and 3, Figs. 3a–d and 4a–d). Modafinil reduced the ED_50_ values of VPA from 328.6 ± 13.17 to 245.3 ± 10.45 mg kg^−1^ and 279.1 ± 16.12 mg kg^−1^ (*F*[3,140] = 5.43; *P* < 0.002) when administered at doses of 50 and 25 mg kg^−1^, respectively (Table 2, Fig. 3a–d). Sulfone metabolite reduced the ED_50_ values of VPA from 328.6 ± 13.17 to 226.4 ± 19.75 mg kg^−1^ and 262.7 ± 10.73 mg kg^−1^ (*F*[3,92] = 9.35; *P* < 0.0001; Table 3, Fig. 4a–d) when administered at doses of 50 and 25 mg kg^−1^, respectively. Acid metabolite when coadministered with VPA only at the dose of 50 mg kg^−1^ significantly enhanced the anticonvulsant activity of VPA by reducing its ED_50_ values from 328.6 ± 13.17 to 241.6 ± 14.52 mg kg^−1^ (*F*[2,101] = 7.94; *P* = 0.0006; Table 4, Fig. 5a–d). Neither modafinil nor its metabolites significantly alter the anticonvulsant action of PB in the MES test in mice (Tables 2–4, Figs. 3a–d, 4a–d and 5a–d). By comparison, GBR 12909 (25 mg kg^−1^) when coadministered with CBZ, PB, PHT, and VPA significantly enhanced the anticonvulsant action of the latter drugs in the MES test by reducing the ED_50_ values of CBZ from 15.53 ± 1.04 to 11.15 ± 1.21 mg kg^−1^ (*F*[2,77] = 4.004; *P* = 0.02), PB from 26.90 ± 2.15 to 15.36 ± 2.71 mg kg^−1^ (*F*[2,61] = 4.571; *P* = 0.01), PHT from 12.10 ± 1.20 to 6.98 ± 0.98 mg kg^−1^ (*F*[2,69] = 5.892; *P* = 0.004), VPA from 286.7 ± 20.11 to 211.4 ± 11.38 mg kg^−1^ (*F*[2,61] = 4.535; *P* = 0.02) (Table 5, Fig. 6a–d). GBR 12909 at the dose of 12.5 mg kg^−1^ did not significantly influence the effects of any tested AEDs (Table 5, Fig. 6a–d).

**Table 2:** Effects of modafinil (MOD) on the anticonvulsant action of carbamazepine (CBZ), phenobarbital (PB), phenytoin (PHT), and valproate (VPA) in the mouse maximal electroshock (MES)-induced seizure model

Treatment (mg kg^−1^)	ED_50_ (mg kg^−1^)	*n*
CBZ + vehicle	11.23 ± 1.20	16
CBZ + MOD (25)	7.48 ± 0.75	16
CBZ + MOD (50)	4.28 ± 0.93***	32
*F* (2; 61) = 11.95; *P* < 0.0001		
PB + vehicle	20.80 ± 3.12	24
PB + MOD (25)	19.40 ± 2.20	24
PB + MOD (50)	13.04 ± 1.76	16
*F* (2; 61) = 2.157; *P* = 0.1244		
PHT + vehicle	11.00 ± 0.93	16
PHT + MOD (25)	8.44 ± 0.84	16
PHT + MOD (50)	5.35 ± 0.86***	32
*F* (2; 61) = 9.831; *P* = 0.0002		
VPA + vehicle	328.6 ± 13.17	40
VPA + MOD (12.5)	303.5 ± 13.19	40
VPA + MOD (25)	279.1 ± 16.12*	40
VPA + MOD (50)	245.3 ± 10.45**	24
*F* (3; 140) = 5.432, *P* = 0.0015		

Results are presented as median effective doses (ED_50_ in mg kg^−1^ ± S.E.M.) of AEDs, protecting 50 % of animals tested against MES-induced hind limb extension. The AEDs were administered i.p. CBZ 30 min, PHT 120 min, PB 60 min, and VPA 30 min prior to the MES test. MOD was administered i.p. at 30 min before the MES test. Statistical analysis of data was performed with one-way ANOVA followed by the post hoc Tukey-Kramer test for multiple comparisons. *n* total number of animals at those doses, whose anticonvulsant effects ranged between 4th and 6th probit (16 and 84 %) according to Litchfield and Wilcoxon (1949)

*F* F-statistics from one-way ANOVA, *P* probability value from one-way ANOVA

*P < 0.05, **P < 0.01, and ***P < 0.001 vs the respective control group (AED + vehicle-treated animals)

**Table 3:** Effects of sulfone metabolite of modafinil (SULF) on the anticonvulsant action of carbamazepine (CBZ), phenytoin (PHT), phenobarbital (PB), and valproate (VPA) in the mouse maximal electroshock (MES)-induced seizure model

Treatment (mg kg^−1^)	ED_50_ (mg kg^−1^)	*n*
CBZ + vehicle	11.23 ± 1.20	16
CBZ + SULF (25)	8.30 ± 0.73	16
CBZ + SULF (50)	5.74 ± 0.82***	24
*F* (2; 53) = 9.086; *P* = 0.0004		
PB + vehicle	20.80 ± 3.12	24
PB + SULF (25)	18.17 ± 1.80	16
PB + SULF (50)	13.97 ± 2.09	8
*F* (2; 45) = 1.017; *P* = 0.3700		
PHT + vehicle	11.00 ± 0.93	16
PHT + SULF (25)	8.27 ± 0.72	16
PHT + SULF (50)	5.95 ± 0.80***	32
*F* (2; 61) = 8.763; *P* = 0.0005		
VPA + vehicle	328.6 ± 13.17	40
VPA + SULF (12.5)	274.2 ± 10.86	8
VPA + SULF (25)	262.7 ± 10.73**	32
VPA + SULF (50)	226.4 ± 19.75***	16
*F* (3; 92) = 9.347, *P* < 0.0001		

Results are presented as median effective doses (ED_50_ in mg kg^−1^ ± S.E.M.) of AEDs, protecting 50 % of animals tested against MES-induced hind limb extension. The AEDs were administered i.p. CBZ 30 min, PB 60 min, PHT 120 min, and VPA 30 min prior to the MES test. SULF derivative of modafinil was administered i.p. at 30 min before the MES test. Statistical analysis of data was performed with one-way ANOVA followed by the post hoc Tukey-Kramer test for multiple comparisons. *n* total number of animals used at those doses whose anticonvulsant effects ranged between 4th and 6th probit (16 and 84 %) according to Litchfield and Wilcoxon (1949)

*F* F-statistics from one-way ANOVA, *P* probability value from one-way ANOVA

**P < 0.01 and ***P < 0.001 vs the respective control group (AED + vehicle-treated animals)

**Table 4:** Effects of acid metabolite of modafinil (ACID) on the anticonvulsant action of carbamazepine (CBZ), phenytoin (PHT), phenobarbital (PB), and valproate (VPA) in the mouse maximal electroshock (MES)-induced seizure model

Treatment (mg kg^−1^)	ED_50_ (mg kg^−1^)	*n*
CBZ + vehicle	11.23 ± 1.20	16
CBZ + ACID (25)	10.22 ± 0.80	16
CBZ + ACID (50)	6.40 ± 0.87**	16
*F* (2; 45) = 6.863; *P* = 0.0025		
PB + vehicle	20.80 ± 3.12	24
PB + ACID (25)	20.28 ± 4.05	32
PB + ACID (50)	14.42 ± 2.16	24
*F* (2; 77) = 0.9940; *P* = 0.3748		
PHT + vehicle	11.00 ± 0.93	16
PHT + ACID (25)	9.86 ± 0.86	24
PHT + ACID (50)	6.25 ± 0.71**	16
*F* (2; 53) = 7.244; *P* = 0.0017		
VPA + vehicle	328.6 ± 13.17	40
VPA + ACID (25)	292.3 ± 19.48	32
VPA + ACID (50)	241.6 ± 14.52***	32
*F* (2; 101) = 7.944, *P* = 0.0006		

Results are presented as median effective doses (ED_50_ in mg kg^−1^ ± S.E.M.) of AEDs, protecting 50 % of animals tested against MES-induced hind limb extension. The AEDs were administered i.p. CBZ 30 min, PB 60 min, PHT 120 min, and VPA 30 min prior to the MES test. ACID derivative of modafinil was administered i.p. at 30 min before the MES test. Statistical analysis of data was performed with one-way ANOVA followed by the post hoc Tukey-Kramer test for multiple comparisons. *n* total number of animals used at those doses whose anticonvulsant effects ranged between 4th and 6th probit (16 and 84 %) according to Litchfield and Wilcoxon (1949)

*F* F-statistics from one-way ANOVA, *P* probability value from one-way ANOVA

**P < 0.01 and ***P < 0.001 vs the respective control group (AED + vehicle-treated animals)

**Table 5:** Effects of GBR12909 (GBR) on the anticonvulsant action of carbamazepine (CBZ), phenobarbital (PB), phenytoin (PHT), and valproate (VPA) in the mouse maximal electroshock (MES)-induced seizure model

Treatment (mg kg^−1^)	ED_50_ (mg kg^−1^)	*n*
CBZ + vehicle	15.53 ± 1.04	24
CBZ + GBR (12.5)	13.79 ± 1.02	24
CBZ + GBR (25)	11.15 ± 1.21*	32
*F* (2; 77) = 4.004; *P* = 0.0222		
PB + vehicle	26.90 ± 2.15	16
PB + GBR (12.5)	19.97 ± 2.17	16
PB + GBR (25)	15.36 ± 2.71*	32
*F* (2; 61) = 4.571; *P* = 0.0141		
PHT + vehicle	12.10 ± 1.20	32
PHT + GBR (12.5)	10.22 ± 0.80	16
PHT + GBR (25)	6.98 ± 0.98**	24
*F* (2; 69) = 5.892; *P* = 0.0043		
VPA + vehicle	286.7 ± 20.11	16
VPA + GBR (12.5)	261.4 ± 13.96	32
VPA + GBR (25)	211.4 ± 11.38*	16
*F* (2; 61) = 4.535, *P* = 0.0146		

Results are presented as median effective doses (ED_50_ in mg kg^−1^ ± S.E.M.) of AEDs, protecting 50 % of animals tested against MES-induced hind limb extension. The AEDs were administered i.p. CBZ 30 min, PHT 120 min, PB 60 min, and VPA 30 min prior to the MES test. GBR was administered i.p. at 30 min before the MES test. Statistical analysis of data was performed with one-way ANOVA followed by the post hoc Tukey-Kramer test for multiple comparisons. *n* total number of animals at those doses, whose anticonvulsant effects ranged between 4th and 6th probit (16 and 84 %) according to Litchfield and Wilcoxon (1949)

*F* F-statistics from one-way ANOVA, *P* probability value from one-way ANOVA

*P < 0.05 and **P < 0.01 vs the respective control group (an AED + vehicle-treated animals)

### Effect of modafinil, its metabolites, or GBR 12909 on total brain antiepileptic drug concentrations

As determined by the fluorescence polarization immunoassay method, modafinil (50 mg kg^−1^) did not significantly affect the total brain concentrations of PB or VPA coadministered at the doses of 13 and 245.3 mg kg^−1^, respectively (Table 6). In contrast, modafinil (50 mg kg^−1^) significantly elevated (by 68 %) the total brain concentration of CBZ coadministered at the dose of 4.3 mg kg^−1^ (*P* < 0.05; Table 6). Additionally, modafinil (50 mg kg^−1^) significantly increased (by 47 %) the level of total brain concentration of PHT coadministered at the dose of 5.4 mg kg^−1^ (*P* < 0.05; Table 6). Similarly, both metabolites sulfone and acid significantly elevated the levels of total brain concentrations of CBZ (by 41 % and by 30 %; *P* < 0.05) and PHT (by 30 and 36 %; *P* < 0.05) and had no effect of the brain levels of PB and VPA (Table 6). In comparison, GBR 12909 coadministered with CBZ, PB, PHT, and VPA did not significantly affect the total brain levels of the latter drugs (Table 6).

**Table 6:** Effect of modafinil (MOD), its sulfone (SULF) and acid (ACID) metabolites, and GBR12909 (GBR) on total brain concentrations of classical antiepileptic drugs (AEDs) in mice

Treatment (mg kg^−1^)	Brain concentration (μg g^−1^ of wet brain tissue)
CBZ (4.3) + vehicle	2.41 ± 0.48
CBZ (4.3) + MOD (50)	4.05 ± 0.41*
CBZ (5.7) + vehicle	3.76 ± 0.35
CBZ (5.7) + SULF (50)	5.31 ± 0.45*
CBZ (6.4) + vehicle	4.46 ± 0.31
CBZ (6.4) + ACID (50)	5.83 ± 0.38*
CBZ (11.2) + vehicle	6.66 ± 0.42
CBZ (11.2) + GBR (25)	6.92 ± 0.48
PB (13.0) + vehicle	23.56 ± 1.10
PB (13.0) + MOD (50)	24.46 ± 0.98
PB (14.0) + vehicle	23.71 ± 0.86
PB (14.0) + SULF (50)	24.85 ± 1.12
PB (14.4) + vehicle	25.46 ± 0.84
PB (14.4) + ACID (50)	26.16 ± 1.28
PB (15.4) + vehicle	24.18 ± 0.70
PB (15.4) + GBR (25)	26.23 ± 0.86
PHT (5.4) + vehicle	1.39 ± 0.20
PHT (5.4) + MOD (50)	2.04 ± 0.22 *
PHT (6.0) + vehicle	1.65 ± 0.19
PHT (6.0) + SULF (50)	2.34 ± 0.23 *
PHT (6.3) + vehicle	1.70 ± 0.15
PHT (6.3) + ACID (50)	2.32 ± 0.20 *
PHT (7.0) + vehicle	3.22 ± 0.30
PHT (7.0) + GBR (25)	3.54 ± 0.24
VPA (211.4) + vehicle	173.66 ± 10.36
VPA (211.4) + GBR (25)	192.47 ± 13.26
VPA (226.4) + vehicle	276.30 ± 11.55
VPA (226.4) + SULF (50)	302.16 ± 18.18
VPA (241.6) + vehicle	346.98 ± 16.10
VPA (241.6) + ACID (50)	369.86 ± 18.14
VPA (245.3) + vehicle	299.73 ± 15.09
VPA (245.3) + MOD (50)	316.90 ± 18.70

Data are presented as mean concentrations (in μg g^−1^ ± S.E.M.) for *n* = 8 mice/group. Total brain concentrations of classical AEDs were quantified using a fluorescence polarization immunoassay technique. Data were statistically verified by using the unpaired Student’s *t* test. All drugs were administered i.p. at doses corresponding to the ED_50_ value from the MES-induced seizures. For more detail, see the legend of Tables 2–5.

*P < 0.05 vs the respective control group (AED-treated animals)

### Effects of modafinil, its metabolites, or GBR 12909, alone and in combination with various antiepileptic drugs, on motor coordination, passive avoidance performance, and muscular strength

Modafinil administered alone at a dose of 50 mg kg^−1^ did not affect motor coordination in mice subjected to the chimney test (Table 7). Similarly, modafinil at 50 mg kg^−1^ did not affect muscular strength in the grip strength test or alter performance in mice challenged with the step-through passive avoidance task (Table 7). When modafinil (50 mg kg^−1^) was administered in combination with CBZ, PB, PHT, or VPA at doses corresponding to their ED_50_ values from the MES test, motor performance in the chimney test, skeletal muscular strength in the grip strength test, and performance in the passive avoidance task were not significantly affected (Table 7). Similarly, sulfone and acid metabolites, as well as GBR 12909 alone or in combination with AEDs, had no significant impact on motor coordination, passive avoidance performance, or muscular strength (Table 7).

**Table 7:** Effects of modafinil (MOD), its sulfone (SULF), and acid (ACID) metabolites, GBR12909 (GBR) and their combinations with classical antiepileptic drugs (AEDs) on passive avoidance performance, muscular strength, and motor performance in mice

Treatment (mg kg^−1^)	Retention time (s)	Grip strength (*N*)	Motor coordination impairment
Vehicle	180 (180; 180)	0.917 ± 0.054	0/8
MOD (50) + vehicle	180 (123.5; 180)	0.908 ± 0.053	0/8
CBZ (4.3) + MOD (50)	180 (123.5; 180)	0.915 ± 0.057	0/8
PB (13.0) + MOD (50)	180 (140; 180)	0.896 ± 0.050	0/8
PHT (5.4) + MOD (50)	180 (125; 180)	0.926 ± 0.055	0/8
VPA (245.3) + MOD (50)	164.5 (110.5; 180)	0.903 ± 0.051	1/8
Vehicle	180 (180; 180)	0.987 ± 0.046	0/8
SULF (50) + vehicle	180 (180; 180)	0.957 ± 0.058	0/8
CBZ (5.7) + SULF (50)	180 (180; 180)	0.943 ± 0.053	1/8
PB (14.0) + SULF (50)	180 (180; 180)	0.951 ± 0.046	0/8
PHT (6.0) + SULF (50)	180 (180; 180)	0.930 ± 0.049	0/8
VPA (226.4) + SULF (50)	180 (125.8; 180)	0.895 ± 0.050	1/8
Vehicle	180 (180; 180)	0.987 ± 0.046	0/8
ACID (50) + vehicle	180 (133.5; 180)	0.961 ± 0.050	0/8
CBZ (6.4) + ACID (50)	180 (180; 180)	0.929 ± 0.049	1/8
PB (14.4) + ACID (50)	180 (170; 180)	0.922 ± 0.051	0/8
PHT (6.3) + ACID (50)	180 (180; 180)	0.955 ± 0.062	0/8
VPA (241.6) + ACID (50)	175.5 (112.5; 180)	0.902 ± 0.060	1/8
Vehicle	180 (180; 180)	0.987 ± 0.046	0/8
GBR (25) + vehicle	180 (133.5; 180)	0.937 ± 0.042	1/8
CBZ (11.2) + GBR (25)	180 (180; 180)	0.979 ± 0.040	2/8
PB (15.4) + GBR (25)	180 (170.5; 180)	0.953 ± 0.059	1/8
PHT (7.0) + GBR (25)	180 (180; 180)	0.944 ± 0.057	1/8
VPA (211.4) + GBR (25)	173.5 (112.5; 180)	0.935 ± 0.053	1/8

Results are presented as follows: (1) median retention times (in seconds; with 25th and 75th percentiles in parentheses) from the passive avoidance task, assessing passive avoidance performance in mice; (2) mean grip strengths (in newtons ± S.E.M.) from the grip strength test, assessing muscular strength in mice; and (3) number of animals showing motor coordination impairment in the chimney test in mice for *n* = 8 mice/group. Statistical analysis of data from the passive avoidance task was performed with nonparametric Kruskal-Wallis ANOVA test, whereas those from the grip strength test were analyzed with one-way ANOVA. The Fisher’s exact probability test was used to analyze the results from the chimney test. All drugs were administered i.p. at times scheduled from the MES test and at doses corresponding to their ED_50_ values against MES-induced seizures in mice

## Discussion

Antiepileptic effects of modafinil and its metabolites have been previously reported in animal studies which examined MES and chemoconvulsant PTZ seizure models (Chatterjie et al. 2004; Chen et al. 2007). The major purpose of this study was to further characterize the anticonvulsant effects of modafinil and its two metabolites when administered alone and in combination with four classical AEDs. We found that modafinil and its metabolites elevated thresholds for electroconvulsions in mice, and the same compounds decreased the ED_50_ values of classical AEDs in the mouse MES model. Because modafinil is known to be a DAT inhibitor that increases extracellular dopamine (Minzenberg and Carter 2008; Zolkowska et al. 2009), we examined the effects of GBR 12909, a prototypical DAT inhibitor. GBR 12909 had effects that were similar to modafinil and its metabolites. Importantly, none of the test compounds affected pharmacokinetics of PB and VPA when given in combination. Intraperitoneal injection of 75 mg kg^−1^ of modafinil or its metabolites significantly elevated the threshold for electroconvulsions in mice, whereas 50 mg kg^−1^ of each compound enhanced the anticonvulsant activity of CBZ, PHT, and VPA, but not that of PB. A 25-mg kg^−1^ dose of modafinil or the sulfone metabolite significantly enhanced the anticonvulsant activity of VPA. Intraperitoneal injection of the DAT blocker GBR 12909 at 25 mg kg^−1^ had similar effects on the activity of all tested AEDs. None of the tested compounds, alone or in combination with AEDs, produced adverse effects.

In the present study, we used linear regression analysis to unequivocally assess the anticonvulsant potential of modafinil, its sulfone, and acid metabolites and GBR in the MEST test in mice. In this seizure model, determining TID_20_ values (i.e., dose that increases threshold by 20 %) allowed a direct comparison of the anticonvulsant potency of the tested compounds. Assessment of the dose-response relationship with linear regression is a standard and common procedure in pharmacological studies, especially in those assessing the anticonvulsant potential of drugs or agents (Löscher et al. 1991; Loscher and Wauquier 1996; Swinyard et al. 1952). With linear regression analysis, it was found that both sulfone and acid metabolites of modafinil produced anticonvulsant effects in doses lower than those of the parent drug modafinil. Since the TID_20_ values for sulfone (23.57 mg kg^−1^) and acid (23.47 mg kg^−1^) metabolites of modafinil were lower than the TID_20_ value for modafinil (49.12 mg kg^−1^), one can ascertain that both metabolites are more favorable with respect to their anticonvulsant potency in the MEST test than modafinil. Of note, the calculation of TID_20_ values allows for a direct comparison of the anticonvulsant effects exerted by the drugs in the MEST test (Löscher et al. 1991; Luszczki and Czuczwar 2005, 2007; Luszczki et al. 2013; Swinyard et al. 1952).

Considering the results from our study (based on the TID_20_ values as determined in the MEST test), it is surprising that a previous study reported modafinil administered alone in a dose of 300 mg kg^−1^ (i.p.) exerts 100 % protection against MES-induced seizures, while sulfone and acid metabolites of modafinil used in the same dose protects 50 and 0 % of animals in the screening mouse MES test, respectively (Chatterjie et al. 2004). Perhaps, doses of both metabolites used in the mouse MES model (300 mg kg^−1^) were too high and thus produced acute adverse effects that disturbed evaluation of their anticonvulsant effects in the MES test. On the other hand, in our study, it was found that the TID_20_ values for sulfone and acid metabolites of modafinil are almost identical suggesting that in low doses, these compounds should produce the same anticonvulsant effects. On the contrary, as reported by Chatterjie et al. (2004), sulfone metabolite in the dose of 300 mg kg^−1^ exerted a 50 % protection in mice subjected to the MES test, whereas the acid metabolite in the dose of 300 mg kg^−1^ produced no anticonvulsant effects in this seizure model. This fact suggests that there may be differences between these metabolites when used in high doses. Nevertheless, more advanced studies are required to investigate this difference between the effects exerted by sulfone and acid metabolites of modafinil.

It is important to note that in our study, modafinil administered systemically (i.p.) increased, in a dose-dependent manner (12.5–75 mg kg^−1^), the threshold for electroconvulsions in the MEST test in mice. Chen et al. (2007) reported that modafinil administered i.p. produced anticonvulsant activity against electroconvulsions in mice. More specifically, the authors documented that modafinil administered i.p. in doses of 22.5, 45, 90, and 180 mg kg^−1^ protected 50, 70, 90, and 70 % of the mice subjected to electroconvulsions, respectively. The apparent discrepancy in potency between the results from our study and those reported earlier by Chen et al. (2007) may be explained either by different seizure models or mouse strains used in experiments. Of note, we applied a current (sine-wave, 0.2-s stimulus duration, 500 V, 25 mA, and 50 Hz) that was almost identical to that used by Swinyard et al. (1952), whereas Chen et al. (2007) have used a nonstandard current (0.4-s stimulus duration and 70 mA) that may produce a different type of electroconvulsions. Additionally, in our study, we used male Albino Swiss (BALB/c) mice, whereas Chen et al. (2007) used male Kunming mice, a strain that is not genetically homogenous, and experimental data indicate that results obtained using this mouse strain may significantly differ from other strains (Shang et al. 2009). Thus, a possible explanation for the more potent anticonvulsant activity of modafinil observed by Chen et al. (2007) is that the Kunming strain of mice is more sensitive to modafinil than the BALB/c strain of mice used in our study. Although this hypothesis is speculative, it can readily explain the observed difference between the results presented in our study for modafinil in the MEST test and those observed by Chen et al. (2007) against electroconvulsions.

The present pharmacokinetic results revealed that modafinil and its metabolites (50 mg kg^−1^) did not alter total brain concentrations of PB and VPA but did elevate concentrations of CBZ and PHT. Results presented by Robertson and Hellriegel (2003) suggest that clinically significant drug-drug interactions with modafinil are most likely connected to effects on two hepatic enzymes: CYP3A4/5, which is the most prevalent human CYP enzyme, and CYP2C9. Studies using human liver microsomes revealed that modafinil can cause weak inhibition of CYP3A4/5 (Robertson and Hellriegel 2003). Such interaction may provide an explanation for increased brain levels of CBZ in combinations with modafinil or its metabolites, since CBZ is primarily metabolized by CYP3A4. Additionally, modafinil and its sulfone metabolite inhibit CYP2C9 in human hepatocytes (Robertson and Hellriegel 2003), and 2C isoforms are major catalysts of PHT metabolism in humans (Cuttle et al. 2000).

The MEST test is used to determine the anticonvulsant potential of a variety of compounds, while the mouse MES model allows evaluation of the effects of tested substances on the activity of classical and second-generation AEDs with proven effectiveness in humans (Löscher et al. 1991). Our results indicate that modafinil and its metabolites elevated, in a dose-dependent manner, the threshold for electroconvulsions in mice. It is important to note that the dose of modafinil providing protection in the MEST model shown here (i.e., 75 mg kg^−1^) is below the dose necessary to stimulate robust motor activity. By contrast, the doses of GBR 12909 providing protection are in the range of motor stimulant doses. For example, Paterson et al. (2010) showed that 150 mg kg^−1^ of modafinil and 15 mg kg^−1^ of GBR 12909 elicit equivalent locomotor activation in mice. Thus, it seems that anticonvulsant effects of modafinil described herein may not be related solely to changes in central dopaminergic activity.

Modafinil and its metabolites at the subprotective dose of 50 mg kg^−1^ (the dose that by itself did not significantly increase the threshold for electroconvulsions) potentiated the anticonvulsant activity of CBZ, PHT, and VPA against MES-induced seizures in mice. By contrast, modafinil and its metabolites at the subprotective dose had no significant impact on the protective action of PB in the mouse MES model. The comparator drug GBR 12909 at the dose of 50 mg kg^−1^ significantly elevated the threshold for electroconvulsions in the MEST test, and in a subprotective dose of 25 mg kg^−1^ potentiated the anticonvulsant activity of CBZ, PB, PHT, and VPA against MES-induced seizures in mice. Chatterjie et al. (2004) reported that modafinil and its metabolites at 30 mg kg^−1^ provide protection against MES-induced seizures with effectiveness ranging from 50 to 25 % after intraperitoneal application in rats. In mice, modafinil administered intraperitoneally at a dose of 300 mg kg^−1^ protected 100 % of animals against MES-induced seizures, while sulfone metabolite provided only a 50 % protection and intermediate acid was toxic at that dose (Chatterjie et al. 2004).

Previous literature implicates the involvement of norepinephrine rather than dopamine in seizure activity. Pretreatment with alpha-1 receptor antagonist terazosin reverses the anticonvulsant effect of modafinil in the MES model, while dopamine receptor (D1 and D2) antagonists have no effect (Chen et al. 2007), which confirms the involvement of noradrenergic system. Catecholamine systems, in particular norepinephrine, are implicated in modulating seizure susceptibility in many animal models. In rodent seizure models, alpha-1 receptor agonists typically exert anticonvulsant effect, while alpha-2 antagonists have proconvulsant activity (Weinshenker and Szot 2002). Many clinically used antiepileptic therapies significantly affect noradrenergic transmission. Classical AEDs like CBZ, PHT, and VPA increase norepinephrine brain levels (Baf et al. 1994a, 1994b; Meshkibaf et al. 1995; Sands et al. 2000). Moreover, genetically altered animals that lack functional noradrenergic systems have lower seizure thresholds and the anticonvulsant effects of PB, PHT, and ketogenic diet are abolished (Krahl et al. 1998; Szot et al. 2001; Waller and Buterbaugh 1985).

The anticonvulsant effects of modafinil plus VPA combinations are pharmacodynamic in nature because modafinil did not significantly alter total brain VPA concentrations in experimental animals. Although modafinil and its metabolites elevated the total brain CBZ and PHT concentrations by a pharmacokinetic mechanism in experimental animals, such combinations did not produce any negative side effects. Of note, total brain AED concentrations were verified in this study with fluorescence polarization immunoassay technique because, as reported earlier, only total brain AED concentrations provide the exact classification and characterization of interactions between AEDs in preclinical studies (Cadart et al. 2002; Luszczki et al. 2003a).

It is worth mentioning that acute administration of AEDs, either alone or in combination with other drugs, can induce adverse effects in experimental animals. For instance, it has been reported that some AEDs, administered systemically (i.p.), produce dose-dependent reductions in skeletal muscular strength in mice (Zadrozniak et al. 2009). With regard to the evaluation of acute adverse effects in the chimney test, it has been documented that the combination of tiagabine with VPA (at doses corresponding to the ED_50_ values from the MES test) causes acute impairment of motor coordination in mice (Luszczki et al. 2003a). Additionally, it has been reported that some AEDs (including, vigabatrin, tiagabine, gabapentin, and pregabalin) administered alone, at doses effective in the MEST test, significantly impair performance in mice subjected to the standard variant of the step-through passive avoidance task (Luszczki et al. 2003b, 2005). Of note, the step-through passive avoidance task provides information about the ability of the tested animals to acquire and retrieve memory (Venault et al. 1986). However, passive avoidance performance can be confounded by a wide range of noncognitive factors, including pain threshold, motivation, emotionality, and motor function (Luszczki et al. 2003b, 2005; Podhorna and Brown 2002). Therefore, in the context of the present study, the passive avoidance test was used as a rapid screening tool for possible side effects, rather than as a pure memory test.

Based on the aforementioned information, it can be assumed that all the behavioral tests performed in this study (i.e., chimney test, passive avoidance task, and grip strength test) are sensitive enough to detect any possible acute adverse effects in animals receiving the combinations of modafinil, its metabolites, and GBR12909 with classical AEDs. The lack of any significant changes in normal behavior in mice exposed to the tested compounds (i.e., modafinil, its metabolites, and GBR12909), alone or in combination with AEDs, shows that these drug treatments produce no measurable adverse effects. Importantly, acute side effects depend on doses of the tested compounds; so, higher doses could induce adverse effects in mice.

In conclusion, our study suggests that the coadministration of modafinil with classical AEDs might be a promising treatment when applied in clinical settings, especially in patients with tonic-clonic seizures or partial convulsions with or without secondary generalization. Sedation and cognitive dysfunction caused by antiepileptic treatments render patients with epilepsy prone to fatigue and excessive daytime sleepiness. Concerns over possible increased risk for seizures have precluded the use of modafinil and other stimulants in patients with epilepsy (Artsy et al. 2012). However, a recent retrospective study demonstrated that epileptic patients taking modafinil for over 10 years had no exacerbation of seizures (Artsy et al. 2012). Our study is first to report the positive effect of modafinil and its metabolites on seizure threshold in rodents. Moreover, our data provide pivotal information on the beneficial effect of modafinil in combination with classical AEDs with no exacerbation of side effects. Our data suggest that further neurochemical and electrophysiological studies are warranted to confirm that modafinil might be a safe and efficacious supplementary therapeutic agent in epilepsy treatment.

## Abbreviations

AEDs: Antiepileptic drugs
MES: Maximal electroshock
MEST: Maximal electroshock seizure threshold
CBZ: Carbamazepine
PB: Phenobarbital
PHT: Phenytoin
VPA: Valproate
DAT: Dopamine transporter
PTZ: Pentylenetetrazole
GBR 12909: 1-[2-[Bis(4-fluorophenyl)methoxy]ethyl]-4-(3-phenylpropyl)piperazine
ANOVA: Analysis of variance
